# Gut microbiota and plasma cytokine levels in patients with attention-deficit/hyperactivity disorder

**DOI:** 10.1038/s41398-022-01844-x

**Published:** 2022-02-23

**Authors:** Liang-Jen Wang, Sung-Chou Li, Shiao-Wen Li, Ho-Chang Kuo, Sheng-Yu Lee, Lien-Hung Huang, Chia-Yin Chin, Chia-Yu Yang

**Affiliations:** 1 Department of Child and Adolescent Psychiatry, Kaohsiung Chang Gung Memorial Hospital and Chang Gung University College of Medicine, Kaohsiung, Taiwan; 2 Genomics and Proteomics Core Laboratory, Department of Medical Research, Kaohsiung Chang Gung Memorial Hospital and Chang Gung University College of Medicine, Kaohsiung, Taiwan; 3 Department of Microbiology and Immunology/Molecular Medicine Research Center, Chang Gung University, Taoyuan, Taiwan; 4 Department of Otolaryngology Head and Neck Surgery, Chang Gung Memorial Hospital, Taoyuan, Taiwan; 5 Department of Pediatrics, Kaohsiung Chang Gung Memorial Hospital and Chang Gung University College of Medicine, Kaohsiung, Taiwan; 6 Kawasaki Disease Center, Kaohsiung Chang Gung Memorial Hospital, Kaohsiung, Taiwan; 7 Department of Psychiatry, Kaohsiung Veterans General Hospital, Kaohsiung, Taiwan; 8 Department of Psychiatry, College of Medicine, Kaohsiung Medical University, Kaohsiung, Taiwan

**Keywords:** Diagnostic markers, Physiology

## Abstract

Attention-deficit/hyperactivity disorder (ADHD) is a common childhood mental disorder with undetermined pathophysiological mechanisms. The gut microbiota and immunological dysfunction may influence brain functions and social behaviours. In the current study, we aimed to explore the correlation of gut microbiome imbalance and inflammation in the pathophysiology of ADHD. Forty-one children with ADHD and thirty-nine healthy-control (HC) individuals were recruited. Faecal samples from all participants were collected and submitted for 16 S rRNA V3–V4 amplicon microbiome sequencing analysis. The plasma levels of 10 cytokines, including TNF-α, IL-6, IL-1β, IL-2, IL-10, IL-13, IL-17A, IFN-α2, IFN-γ, and MCP-1, were determined using a custom-made sandwich enzyme-linked immunosorbent assay (ELISA) developed by Luminex Flowmetrix. There was no significant difference between the ADHD and HC groups in species diversity in the faeces, as determined with α-diversity and β-diversity analysis. In the ADHD group, three differentially abundant taxonomic clades at the genus level were observed, namely *Agathobacter*, *Anaerostipes*, and *Lachnospiraceae*. Top differentially abundant bacteria and representative biological pathways were identified in children with ADHD using linear discriminant analysis (LDA) effect size (LEfSe), and the phylogenetic investigation of communities by reconstruction of unobserved states (PICRUSt) analysis, respectively. The plasma levels of TNF-α were significantly lower in children with ADHD than in HCs. Within the ADHD group, the levels of TNF-α were negatively correlated with ADHD symptoms and diversity of the gut microbiome. Our study provides new insights into the association between gut microbiome dysbiosis and immune dysregulation, which may contribute to the pathophysiology of ADHD.

## Introduction

Children with attention-deficit/hyperactivity disorder (ADHD) suffer from failures in academic performance and interpersonal relationships, and from multiple psychiatric comorbidities, which, without proper interventions, may persist into adulthood [1]. Although multiple parameters may be involved in ADHD pathophysiology, a major contributing factor is the dysfunctionality of the ‘gut–brain axis’—which refers to the link between intestinal function, gut microbiota and the central nervous system—during the course of ADHD development, due to gut microbial dysbiosis [2–4]. However, a systematic review that investigated the role of dysbiosis in neurodevelopmental disorders was inconclusive due to the marked heterogeneity between different studies [5]. Therefore, a thorough investigation of the gut microbiota profiles in children with ADHD may shed new light on its underlying pathophysiology [6–11].

To analyse the phylogeny and taxonomy of samples with complex microbiomes or from complex environments, 16 S ribosomal RNA (rRNA) sequencing is commonly used for amplicon sequencing [12]. Although microbiota analyses with case-control design are exploratory, they establish the basis of longitudinal studies to clarify the pathogenesis of ADHD [13]. One previous study found significantly decreased microbial diversity (α-diversity) in patients with ADHD compared with controls [14]. A study using the classical gene-set enrichment analysis approach found an association between ADHD and the genus *Desulfovibrio*, order *Clostridiales* [15]. Our research group previously found that the abundance of *Bacteroides* and *Sutterella* was different between children with ADHD and HCs [16]. Studies on the potential significance of *Bifidobacterium*, which is involved in the dopamine neural reward system, suggested that *Bifidobacterium* abundance may serve as a biomarker for ADHD [17, 18]. Moreover, *Faecalibacterium*, a genus of the *Ruminococcaceae* family, *Anaerotaenia* and *Gracilibacter* at the genus levels have been associated with attention deficit, which indicates that dysbiosis in gut microbiota is potentially involved in the pathophysiology of ADHD [19–21]. Based on these findings, we hypothesised that the gut microbiota imbalance may cause changes in the gut–brain axis and further affect neurotransmitter levels, which contribute to the clinical presentation of ADHD [16]. However, published studies have reported inconclusive results.

The intestinal microbiota may influence the development of multiple organs, including the brain, lungs and the immune system and overall body growth [22, 23]. In an animal study, microbiota composition changes were associated with activation of the neuroimmune response [24]. Children’s brains may be susceptible to pathological insult in combination with instability and immaturity of the gut microbiota [25]. The microbiome and the released factors in the blood may stimulate peripheral immune cells, thus influencing the blood–brain barrier (BBB) and other factors in the neurovascular system [26]. Peripheral immune-cell composition is modulated by the microbiome, which may be also associated with brain development and alterations in neurotransmitter systems [27–29].

Imbalanced cytokine levels may represent an important linking mechanism between ADHD and allergies [30, 31]. For example, concentrations of interleukin (IL)-16, IL-13, tumour necrosis factor (TNF)-α, and interferon (IFN)-γ were associated with clinical symptoms and the performance of children with ADHD in the Conner’s Continuous Performance Test (CPT) [32]. In addition, the levels of IFN-γ and IL-13 were slightly higher in children with ADHD than in healthy controls (HCs) [33]. Evidence has also indicated that cytokines affect brain development, particularly the prefrontal cortex and anterior cingulate cortex [34, 35]. Furthermore, TNF-α has been found to influence synaptic development in the hippocampus, whereas IL-1β, IL-6, and IL-10 affect memory function [36]. Serum IL-6 and IL-10 levels were relatively higher in children with ADHD, compared with HCs [37, 38]. A case–control study indicated oxidative stress (glutathione) and immune abnormalities (a trend for lower plasma IL-5 levels) in untreated patients with ADHD [39]. A recent study found a significant correlation between IL-6, TNF-α and hyperactivity/impulsivity symptoms in young people with obesity [40].

We hypothesised that dysbiosis of gut microbiota may be partly associated with the pathogenesis of ADHD. In addition, we proposed that immunological dysregulation is involved in alterations of the gut microbiome. Therefore, our study aimed to explore the correlation between gut microbiome imbalance and inflammatory cytokine levels in the pathophysiology of ADHD.

## Materials and methods

### Study participants

Our study protocol was approved by the Institutional Review Board (IRB) at Chang Gung Memorial Hospital in Taiwan (IRB No. 201702019A3). Children with ADHD were recruited from Chang Gung Children’s Hospital in Taiwan; HCs were recruited from the community close to the hospital. Study protocols were explained to the participants and their parents or guardians prior to their entry into this study, and informed consents were signed by both the participants and their caregivers upon their agreement.

The following inclusion criteria for patients with ADHD were applied: (a) children diagnosed with ADHD, as confirmed by a senior child psychiatrist through structured interviews using the Kiddie-Schedule for Affective Disorder and Schizophrenia-Epidemiological Version (K-SADS-E) [41, 42]; (b) children aged between 6 and 16 years; and (c) children with no prior history of any medical treatment for ADHD (methylphenidate, atomoxetine or clonidine). The following exclusion criteria of this study were applied: (a) children with a history of major neuropsychiatric diseases, including autism-spectrum disorder (ASD), intellectual disabilities, major depressive disorders (MDD), bipolar disorders, psychotic disorders or substance-use disorders (SUD); (b) children with any major physical illnesses, such as epilepsy, neuroendocrine or gastrointestinal disorders; (c) children on a vegetarian diet or those who were administered any anti-inflammatory drugs, antibiotics or probiotics within one month.

The HC group consisted of children without ADHD diagnosis (aged between 6 and 16 years) within the same catchment area. HCs were also interviewed using the K-SADS-E and were not diagnosed with the aforementioned major neuropsychiatric diseases (ASD, intellectual disabilities, MDD, bipolar disorders, psychotic disorders or SUD). Individuals on a vegetarian diet, suffering with major physical illnesses, or receiving any anti-inflammatory drugs, antibiotics or probiotics during recruitment were also excluded.

### Sample collection

Both, children with ADHD and HCs, were asked to collect their faecal samples by scooping a pea-sized piece of faeces in a sterile plastic tube. Samples were stored at 4 °C after collection and transferred to a −80 °C refrigerator in our laboratory within 24 h. Total genomic DNA (gDNA) was extracted from each sample using the QIAamp PowerFecal DNA Kit from Qiagen. The concentration of gDNA was determined with Qubit fluorometric quantitation prior to polymerase-chain reaction (PCR).

### 16S library preparation and paired-end sequencing

The 16S V3–V4 region was amplified by a specific primer pair (319 F: 5′ CCTACGGGNGGCWGCAG 3′, 806 R: 5′ GACTACHVGGGTATCTAATCC 3′). First, faecal gDNA (12.5 ng) was used for V3–V4 region amplification. The KAPA HiFi HotStart Ready Mix (Roche) was used under the following PCR conditions: 95 °C for 3 min; 25 cycles of 95 °C for 30 sec, 55 °C for 30 sec and 72 °C for 30 sec; 72 °C for 5 min. The length of the PCR product was approximately 500 bp. The main fragments were then purified with AMPure XP beads, followed by a second PCR with dual indices and sequencing adapters (Nextera XT Index Kit, Illumina). Finally, equal amounts of the indexed PCR product were denatured and pooled, and then the 16S library was sequenced on an Illumina MiSeq sequencer (paired-end 2 ×300 bp reads).

### Immunological profile

Blood samples were obtained between 7:00 and 8:00 AM. A multiplex bead-based assay was developed using the Luminex Flowmetrix system (Luminex, Austin, TX) to investigate the cytokine levels in the plasma. Each capture antibody for an individual cytokine was coupled to a different bead set (Upstate Biotechnology Beads, NY; Luminex). Recombinant cytokines pre-diluted in pooled blank plasma from HCs were used as standards; test sera from patients or controls were examined with multiplex assays as previously reported [33, 34, 43–45] for several cytokines, including TNF-α, IL-6, IL-1β, IL-2, IL-10, IL-13, IL-17A, IFN-α2, IFN-γ and MCP-1. First, beads were incubated with 50-μl diluted standards or plasma and then with detection antibodies for 2 h at room temperature. Next, mixtures were incubated with biotin as a reporter for 1.5 h, followed by incubation for 30 min with phycoerythrin-conjugated streptavidin. Finally, the concentration of cytokines in the bead array was measured with the fluorescence intensity calculated with the Flowmetrix software.

### Clinical assessments

A senior psychiatrist confirmed the clinical diagnosis of ADHD and neuropsychiatric disorders in the enroled children with ADHD and HCs through face-to-face interviews [41]. K-SADS-E is designed to assess psychiatric disorders in children and adolescents according to The Diagnostic and Statistical Manual of Mental Disorders, Fourth Edition (DSM-IV) criteria using semi-structured interviews [41]. The Chinese version of K-SADS-E has been established as a reliable and valid assessment tool in Taiwan [42]. Furthermore, the Wechsler Intelligence Scale for Children—Fourth Edition (WISC-IV) [46] was conducted by a child psychologist to assess participants’ cognitive function. The psychologist also performed the Conners’ CPT [47] to examine participants’ attention. Participants’ parents and teachers were requested to complete the parent and teacher forms, respectively, of the Swanson, Nolan, and Pelham Version IV Scale (SNAP-IV), to measure ADHD core symptoms at home and school [48–50].

### Statistical and bioinformatics analysis

The sample size was estimated using the software package G-Power 3.1; based on the settings of 80% power, *p* = 0.05. The Statistical Package for the Social Sciences Version 16.0 (SPSS Inc., Chicago, IL, USA) was used for data analysis. Mean ± standard deviation or frequency were presented as variables. The chi-square test was used to analyse the sex distribution between children with ADHD and HCs. The independent t-test was used to determine the differences in demographics, clinical symptoms and neuropsychological function between patients and controls. Two-tailed *p* < 0.05 was regarded as statistically significant.

For the analysis of 16 S rRNA sequencing data, the main workflow was performed using QIIME (v1.9.1) [51], which contains multiple programmes. All raw data (*.fastq) in the data folder were analysed with FastQC (v0.11.5) followed by MultiQC (v0.9). Trimming of primer sequences was performed using Cutadapt. Then, R1 and R2 of paired-end reads were merged using FLASH v1.2.11, and only merged reads were used in further analysis. Low-quality reads (*Q* < 20) were removed using the QIIME pipeline. Chimera sequences were identified using UCHIME v4.2 (with the reference Gold database) [52] to obtain the effective tags. Operational taxonomic unit (OTU) picking was performed using the UPARSE [53] function in the USEARCH pipeline (v.7) [54] with 97% identity [55]. Ideally, the sequences in the same OTUs were from the same group, known as a hypothetical classification unit [56]. The RDP classifier (v2.2) algorithm [57] was adapted to annotate taxonomy classifications based on the SILVA database (SILVA v132 rep_set_16 S_only/99/silva_132_99_16S.fna, rep_set_aligned/99/99_alignment.fna) [56, 58] for each representative sequence, which was established with an 80% minimum confidence threshold to identify an assignment. Species annotation was visualised with KRONA (v2.7) [59]. Moreover, sequencing results were further validated with the DADA2 package followed by standard default settings [60]. Differences of bacterial taxonomy between children with ADHD and HCs were analysed using analysis of covariance (ANCOVA) following adjustment with age and sex. Bonferroni-corrected *p*-values were calculated as 0.05 (alpha error) divided by the number of parameters in each figure.

## Results

### Demographic data

Faecal samples from 41 children with ADHD (mean age: 8.0 years old, 73.2% male) and 39 HC individuals (mean age: 10.0 years old, 56.4% male) were collected. The characteristics of the ADHD and HC groups in our study are summarised in Table 1. In our cohort, higher male-to-female ratios were found in ADHD, which is consistent with the known ADHD prevalence ratio ranging from 2:1 to 10:1 [61].

**Table 1:** Characteristics of patients with ADHD and healthy control children.

	ADHD (*n* = 41)	Controls (*n* = 39)	Statistical values^a^	*p* value
Sex (*n*, %)			2.468	0.116
Boy	30 (73.2)	22 (56.4)		
Girl	11 (26.8)	17 (43.6)		
Age (y)	8.0 ± 1.7	10.0 ± 2.7	−3.859	<0.001*
Height (cm)	128.2 ± 9.4	140.6 ± 15.0	−4.156	<0.001*
Weight (kg)	29.2 ± 9.7	36.4 ± 13.4	−2.576	0.012*
BMI	17.5 ± 4.2	17.8 ± 4.3	−0.315	0.754
Way of delivery			0.150	0.698
Normal spontaneous delivery	30 (73.2)	30 (76.9)		
Cesarean section	11 (26.8)	9 (23.1)		
WISC-IV				
Full Scale Intelligence Quotient	98.8 ± 11.1	108.7 ± 12.9	−3.452	0.001*
Verbal Comprehension Index	102.9 ± 13.7	104.5 ± 12.7	−0.498	0.620
Perceptual Reasoning Index	96.7 ± 11.3	110.4 ± 14.8	−4.371	<0.001*
Working Memory Index	101.3 ± 12.5	109.8 ± 11.1	−3.019	0.004*
Processing Speed Index	94.8 ± 9.2	102.8 ± 10.0	−3.466	0.001*
Conners’ CPT				
Confidence Index	67.8 ± 23.1	47.2 ± 22.9	3.732	<0.001*
Omission	64.0 ± 22.8	52.1 ± 10.1	2.825	0.007*
Commission	50.3 ± 9.1	46.7 ± 10.0	1.584	0.118
Hit Rate	59.9 ± 13.4	55.8 ± 10.6	1.451	0.151
Detectability	51.3 ± 7.2	47.7 ± 10.8	1.617	0.110
Clinical measures				
SNAP-IV parent form (I)	16.4 ± 6.6	4.4 ± 5.5	8.229	<0.001*
SNAP-IV parent form (H)	14.5 ± 7.1	3.1 ± 4.5	8.046	<0.001*
SNAP-IV teacher form (I)	16.0 ± 6.2	3.5 ± 3.8	9.739	<0.001*
SNAP-IV teacher form (H)	12.1 ± 7.3	2.4 ± 2.9	6.857	<0.001*

*Notes:* Data are expressed as mean ± SD or *n* (%); *CPT* continuous performance test, *SNAP-IV* the Swanson, Nolan, and Pelham–Version IV Scale for ADHD; *WISC-IV* the Wechsler Intelligence Scale for Children–Fourth Edition, *I* inattention scores, *H* hyperactivity/impulsivity scores.

^a^Statistical values are expressed as *t*-value or *χ*^2^: *p < 0.05.

### Gut microbiota analyses

The 16S V3–V4 library from faecal samples was constructed and sequenced in the Illumina sequencer platform according to standard procedures. We used a rarefaction curve to estimate gut microbiome species diversity. Rarefied OTU tables were generated and α-diversity metrics were established for each rarefied OTU table. For rarefaction curve generation, a certain amount of sequencing data was randomly selected from the samples to represent the number of species. When the curve became flat, a reasonable sequencing depth was acquired, indicating that more sequencing depth was likely to yield only a few additional species. Based on the chart, both the ADHD and HC samples had sufficient sequencing depth for species identification. The X axis illustrates the number of sequences (per sample), and the Y axis demonstrates the number of observed species (Supplementary Fig. 1). Rarefaction curves showed that a plateau of species richness (up to 200 OTUs) was achieved in approximately 40,000 reads per sample (Supplementary Fig. 1), indicating that the sequencing depth included considerable information regarding total species richness.

**Fig. 1:**
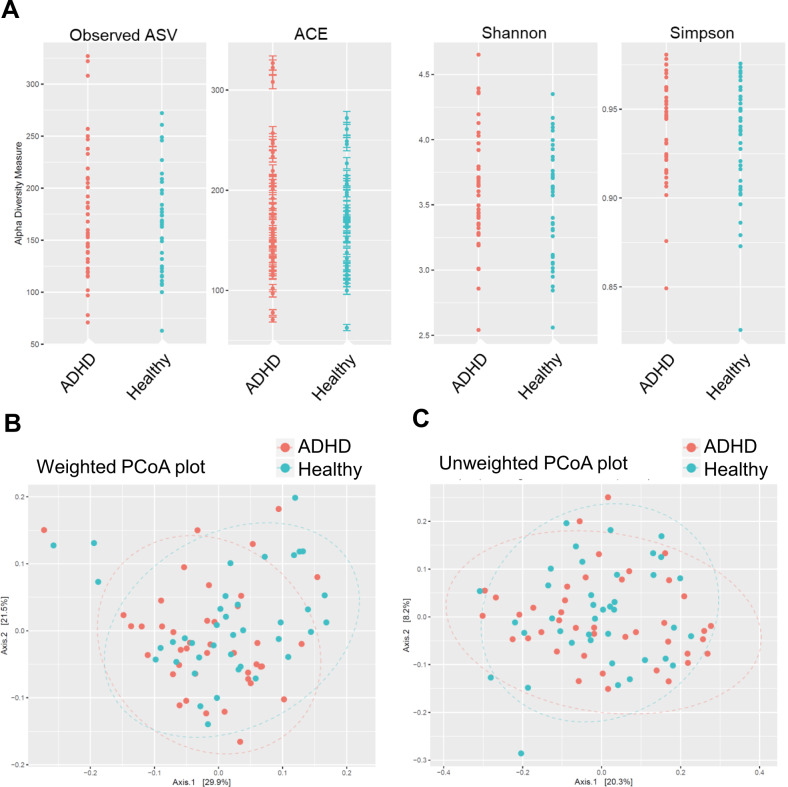
The diversity index and profiling of gut microbiome in patients with ADHD and healthy controls. **A** The ACE richness estimator, Shannon and Simpson index were analysed to determine species richness, and it was found that there was no significant difference between the ADHD and healthy-control groups. **B** The weighted PCoA and **C** unweighted PCoA plot were performed based on the OTU level to evaluate the variation in gut microbiota composition among the groups. β-diversity analysis indicates the extent of similarity between microbial communities.

We further analysed the gut microbiota composition in children with ADHD. The ACE richness estimator, Shannon index and Simpson index were analysed to estimate species richness. Our data showed that no significant difference was observed in the aforementioned indices between the ADHD and HC groups (Fig. 1A). Weighted PCoA and unweighted PCoA plots were performed based on the OTU level to evaluate the variation in gut microbiota composition in both groups (Fig. 1B, C). We also considered sex predilection with respect to gut microbiota. We found that the microbiome community in either male or female participants was similar between ADHD and HC groups (Supplementary Fig. 2). Taken together, α-diversity and β-diversity analyses indicate that the gut microbiome was similar between children in ADHD and HC groups.

**Fig. 2:**
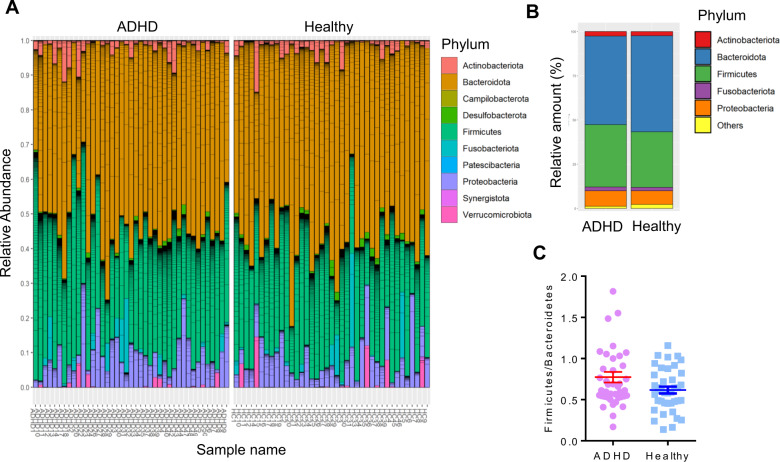
Relative abundance of the most prevalent bacteria in phylum level in patients with ADHD and healthy controls. **A** The relative abundance of the most prevalent bacteria in phylum level in individual ADHD and healthy was shown. **B** The main phylum were *Bacteroidota*, *Firmicutes*, *Proteobacteria*, *Actinobacteriota* and *Fusobacteriota* in gut microbiome. **C** The Firmicutes-to-Bacteroidetes ratio (F/B ratio) in ADHD and HCs. The p-value was determined using analysis of covariance (ANCOVA) following adjustment with age and sex.

Next, the gut microbiome at different phylogenetic levels from phylum to species was comprehensively determined in children with ADHD and HCs. Figure 2 shows the relative representation of the top-5 bacteria at the phylum level in the ADHD and HC groups. Our data showed that the main phyla in the gut were the *Bacteroidotes*, *Firmicutes*, *Proteobacteria*, *Actinobacteriota* and *Fusobacteriota* (Fig. 2A, B). The distribution of the main top-5 phyla was similar in children with ADHD and HCs. The *Firmicutes*-to-*Bacteroidetes* ratio (F/B ratio) showed a slightly increasing trend in children with ADHD compared with HCs (Fig. 2C). Supplementary Fig. 3 illustrates the bacterial taxonomic distributions from class to genus levels in the ADHD and HC groups. The most abundant classes, orders, families and genera were *Bacteroidia*, *Bacteroidales*, *Bacteroidaceae* and *Bacteroides*, respectively. Compared with the HC group, children with ADHD had increased representation of three bacterial genera, including *Agathobacter*, *Anaerostipes* and *Lachnospiraceae UCG-010* (Supplementary Fig. 4). The *p*-value was determined using ANCOVA following adjustment for age and sex. Supplementary Fig. 5 shows the bacterial taxonomic distributions at the species level in the ADHD and HC groups.

**Fig. 3:**
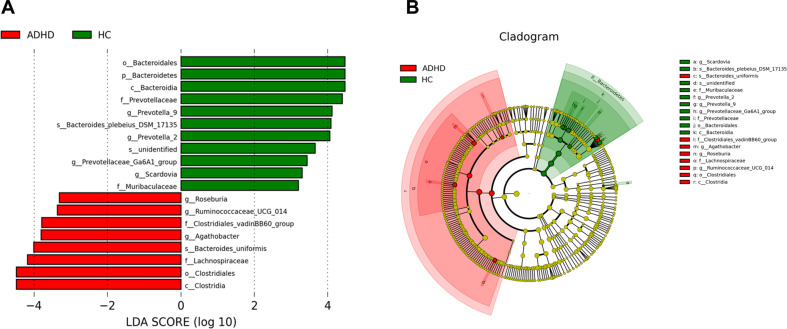
Linear discriminant analysis (LDA) effect size (LEfSe) revealed differentially abundant bacterial taxa in ADHD. **A** LEfSe analysis showed that abundance of bacterial genus was altered as compared between ADHD and HC. **B** Comparison of the taxonomy for ADHD and HC group. The taxonomy was analysed and performed as cladogram. The relative colour represented the more-abundance bacterial taxonomy in each group.

**Fig. 4:**
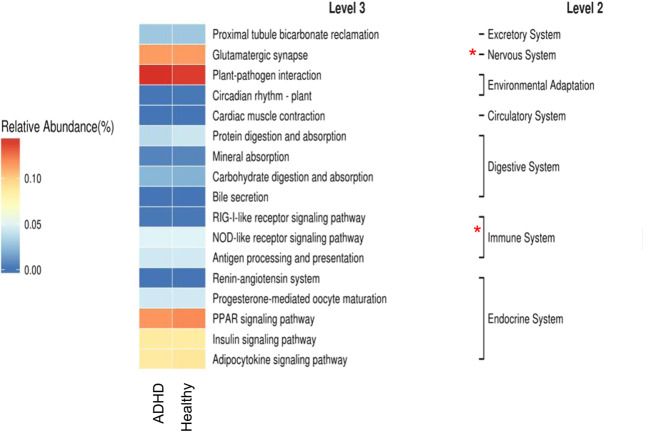
Nervous-system and immune system functions were enriched in ADHD by PICRUSt analysis. The functions of bacterial communities were analysed based on PICRUSt by KEGG annotation at level 2 and level 3.

In addition, we applied the LDA effect-size (LEfSe) algorithm to identify metagenomic biomarkers in children with ADHD. LEfSe analysis revealed differentially abundant bacterial taxa between the ADHD and HC groups (Fig. 3). Our results identified the genera *Roseburia*, *Ruminococcaceae* and *Agathobacter* in the ADHD group, which showed a higher LDA score based on LEfSe, reflecting a significant increase in frequency in the ADHD group compared with HC participants (Fig. 3A, B). Figure 4 demonstrates functional predictions of all samples using the phylogenetic investigation of communities by reconstruction of unobserved states (PICRUSt). Based on the heatmap of the microbiota identified in the HC and ADHD groups, the most representative pathways were proximal tubule bicarbonate reclamation followed by glutamatergic synapses. The nervous and immune systems were also present in the functional prediction of the microbiota. Collectively, the population of some bacteria was enriched in children with ADHD. Altered bacteria composition in ADHD may affect the gut-to-brain axis, thus our PICRUst analysis revealed the most abundant nervous-related pathway in these children. Interestingly, immune-related pathways were also enriched in the functional predictions in our dataset. We then profiled the inflammatory-related cytokines in our cohort.

### Immunological profile

**Fig. 5:**
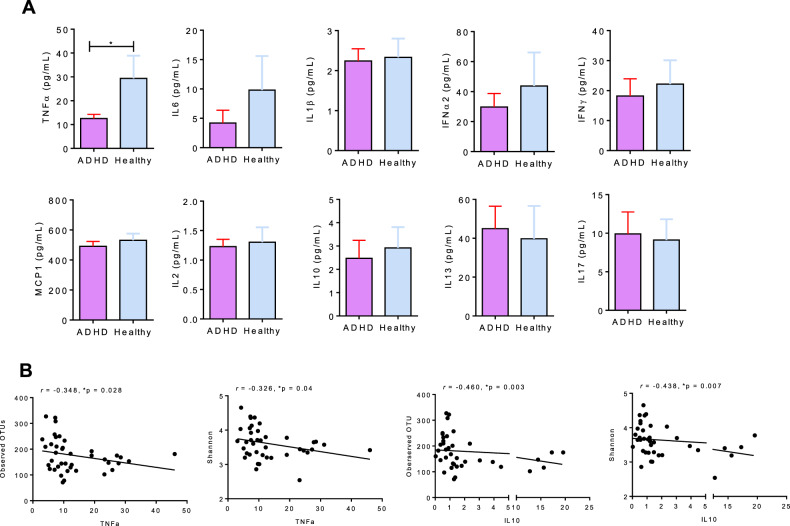
The plasma levels of IFN-α2, IFN-γ, IL-1B, IL-2, IL-10, IL-13, IL-17A, MCP-1 and TNF-α in patients with ADHD and healthy controls. **A** The plasma levels of TNF-α were significantly lower in the ADHD group, compared with the HC group. **B** In the ADHD group, the plasma levels of IL-10 levels were negatively correlated with observed OTU (*r* = -0.460, *p* = 0.003) and Shannon index (*r* = -0.438, *p* = 0.007). The plasma levels of TNF-α levels were negatively correlated with observed OTU (*r* = −0.348, *p* = 0.028) and Shannon index (*r* = −0.326, *p* = 0.04).

The plasma levels of TNF-α were significantly lower in the ADHD group than in the HC group (Fig. 5A). Except for TNF-α, the plasma levels of IL-6, IL-1β, IL-2, IL-10, IL-13, IL-17A, IFN-α2, IFN-γ and MCP-1 were not significantly different between the two groups (Fig. 5A). We further identified the correlation of the cytokine profile with the gut microbiome composition or clinical symptoms in ADHD. In the ADHD group, the plasma levels of TNF-α showed an inverse correlation with the observed OTUs (*r* = −0.348, *p* = 0.028) and Shannon index (*r* = −0.326, *p* = 0.04) (Fig. 5B). The plasma levels of IL-10 also showed an inverse correlation with the observed OTUs (*r* = −0.460, *p* = 0.003) and Shannon index (*r* = −0.438, *p* = 0.007). Moreover, TNF-α levels in children with ADHD were negatively correlated with attention deficit (*r* = −0.436, *p* = 0.004) and hyperactivity/impulsivity symptoms (*r* = −0.376, *p* = 0.015).

## Discussion

In this study, the 16 S rRNA sequencing platform was implemented for the screening of bacterial communities from faecal samples of children with ADHD and HC participants. Species richness in the faeces, which was determined by α-diversity and β-diversity analysis, was not significantly different between the participants of ADHD and HC groups. Our results are in line with a previous case–control study [17] that also used the same metrics (*α*-diversity or relative abundance of *Fusobacterium*). Nonetheless, our data showed that a higher abundance of three genera *Agathobacter*, *Anaerostipes* and *Lachnospiraceae UCG-010* was found in the ADHD group compared with the HC group. Furthermore, our study revealed a slightly higher F/B ratio in children with ADHD than in HCs. The F/B ratio has been widely investigated in previous studies regarding gut microbiota in both humans and mice and has been associated with obesity and other diseases [62].

A previous animal study showed that administration of an anti-mouse IL-6 receptor antibody (MR16-1) significantly improved the decreased F/B ratio and normalised the gut microbiota composition in susceptible mice under social-defeat stress [63]. Another study indicated that the composition of the human gut microbiota, particularly the F/B ratio, varies significantly between different age groups [64]. The relative abundance of the components of the F/B ratio is highly variable because a number of lifestyle-associated factors (i.e., diet, antibiotic consumption and physical activity) potentially influence the microbiota composition in the gastrointestinal tract [62]. Therefore, the mechanism underlying the association between the F/B ratio and ADHD requires further exploration.

In children with ADHD, a total of three differentially abundant taxonomic clades were observed, namely *Agathobacter, Anaerostipes* and *Lachnospiraceae UCG-010*. *Agathobacter* is a bacterial genus from the family *Lachnospiraceae*. The relative abundance of *Agathobacter* has been associated with physician-diagnosed inhalant allergies in school-age children [65] and was associated with ASD and sleep problems [66]. *Anaerostipes* is a Gram-positive anaerobic bacterial genus from the family *Lachnospiraceae*. Products of bacterial metabolism by *Anaerostipes* and *Lachnospiraceae* have been related to the aetiology of depression [67]. Nevertheless, the association between ADHD and these three taxonomic clades has not been reported. Our pathway analyses implied that the nervous and immune systems were involved in the functional prediction of the microbiota in children with ADHD. In our studies, we identified that the amount of *Bacteroides uniformis* was increased in ADHD (Fig. 3A). Nonetheless, it is challenging to use short-read 16 S rDNA-based sequencing technologies for bacterial taxonomy at the species level. In the future, we will expand the cohort and use species-specific primers by quantitative PCR for more precise detection of bacteria at the species level in patients with ADHD.

We found that plasma TNF-α levels were lower in the ADHD group than in the HC group, which is consistent with a previous study [68]. However, another study indicated that TNF-α was neither significantly associated with ADHD diagnosis nor with ADHD symptoms, as assessed by parents [69]. Our findings revealed that the levels of TNF-α were negatively correlated with ADHD symptoms and the diversity of the gut microbiome. Nonetheless, a positive significant correlation between TNF-α levels and ADHD rating scores was observed in another study [40]. In an animal study, anti-TNF-α therapy potentially regulated the gut microbiota and intestinal barrier function, and further ameliorated proteoglycan-induced arthritis [70]. Another study demonstrated that gut microbial dysbiosis is involved in the pathogenesis of Parkinson’s disease (PD) [71]. Mice with PD benefited from faecal microbiota transplantation through reduction of TLR4/TNF-α signalling and suppression of neuroinflammation [71]. Overall, we propose that there may be a link between the gut microbiome, immunological functions and the pathophysiology of ADHD.

Moreover, we did not find an association between ADHD and cytokine levels, other than TNF-α. Many cytokines, including IFN-α, IFN-γ, IL-2, IL-10, IL-1, IL-6 and TNF-α, can cross the BBB and affect nerve development or the function of the hypothalamic–pituitary–adrenal axis [40, 45]. Specifically, an increased level of IL-6 in patients with ADHD was reported in previous studies [37, 68, 69]. The inconsistencies in the findings between our study and previous studies may be related to the small sample size and differences in patient characteristics (environment and allergens). Further evidence is required to clarify whether production of various cytokines may play a role in the pathogenesis of ADHD.

Our study is limited due to the following points. First, our study design was cross-sectional rather than longitudinal; therefore, early life events (mode of birth delivery, history of breastfeeding, previous antibiotic use and dietary patterns) that may impact the initial colonisation and development of microbiota were not available in this study [72, 73]. Second, although we found that TNF-α was significantly correlated with some bacterial taxa in children in the ADHD group, we measured TNF-α only in the plasma, which is not representative of the whole spectrum of immunological functions. The underlying mechanism of interaction between gut microbiota and the immune system still requires further investigation. Third, the age and sex of the recruited children and the HC individuals were not matched. It is possible that the differential abundance of the gut microbiota was confounded by the differences in demographic characteristics. Fourth, faecal samples were not collected at a certain/fixed time during the day. It remains unclear whether gut microbiome taxa are influenced by the various timepoints of the collection [74]. Finally, the gut microbiota composition may vary across different ethnic groups or socioenvironmental circumstances. Caution should be used when comparing out results to those of previous studies. There is heterogeneity between previous studies regarding the sample size, study design, drug-naive status and technology applied for microbiome sequencing and cytokine analysis [3, 75]. Further research is required to determine whether our findings can be extrapolated to other populations.

In summary, a total of three differentially abundant taxonomic clades at the genus level (*Agathobacter*, *Anaerostipes* and *Lachnospiraceae UCG-010*) were observed in children with ADHD. The plasma levels of TNF-α were lower in the ADHD group than in the HC group. Our results indicate that the association between gut microbiome dysbiosis and immune dysregulation may contribute to the pathophysiology of ADHD.

## Supplementary information

Supplementary Fig. 1.

Supplementary Fig. 2.

Supplementary Fig. 3.

Supplementary Fig. 4.

Supplementary Fig. 5.

## Data Availability

The data are available within the paper from the paper corresponding author on reasonable request.
