# Supplementary material for: Gut microbiota and plasma cytokine levels in patients with attention-deficit/hyperactivity disorder
Source: Transl Psychiatry. 2022 Feb 23;12:76. doi: 10.1038/s41398-022-01844-x (PMC8866486; doi:10.1038/s41398-022-01844-x)
Supplement: Supplementary file 1 — Supplementary Fig. 1. [file 41398_2022_1844_MOESM1_ESM.pdf]

Rarefaction curves

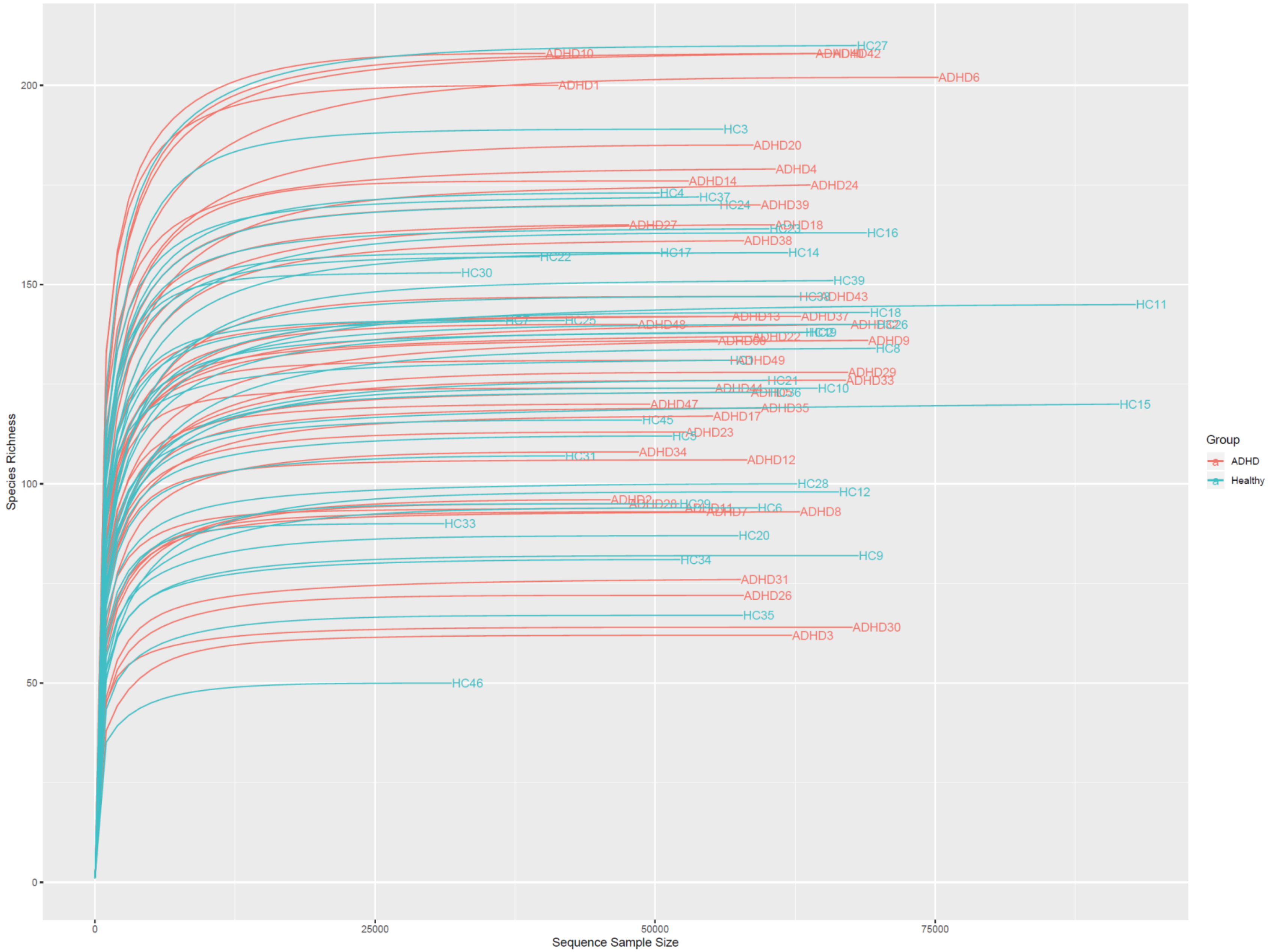

Supplementary Fig. 1. The rarefaction curve of gut microbiome in ADHD and healthy controls.
