# Supplementary material for: Gut microbiota and plasma cytokine levels in patients with attention-deficit/hyperactivity disorder
Source: Transl Psychiatry. 2022 Feb 23;12:76. doi: 10.1038/s41398-022-01844-x (PMC8866486; doi:10.1038/s41398-022-01844-x)
Supplement: Supplementary file 2 — Supplementary Fig. 2. [file 41398_2022_1844_MOESM2_ESM.pdf]

Male

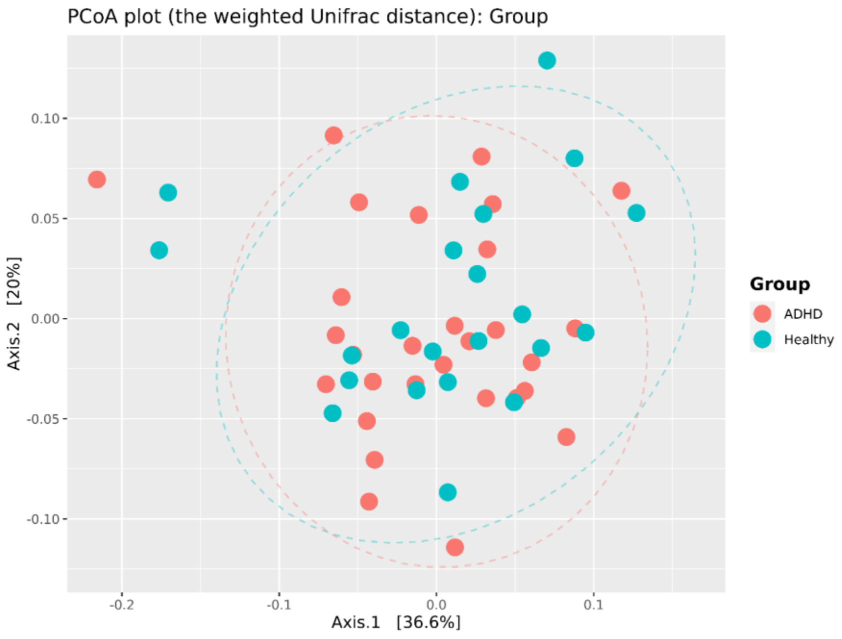

Female

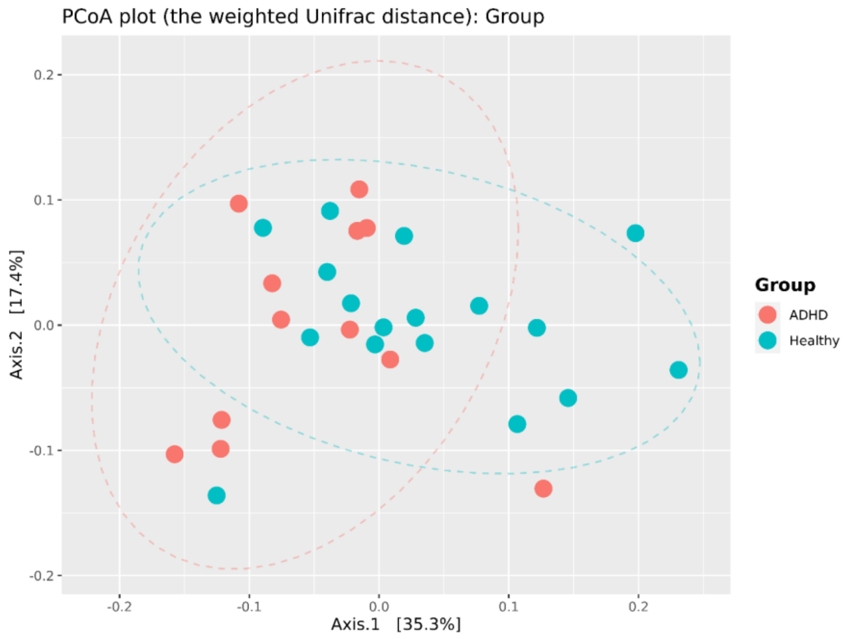

Supplementary Fig. 2. The gut microbiome PCoA plot in male and female ADHD patients and healthy controls.
