# Supplementary material for: Gut microbiota and plasma cytokine levels in patients with attention-deficit/hyperactivity disorder
Source: Transl Psychiatry. 2022 Feb 23;12:76. doi: 10.1038/s41398-022-01844-x (PMC8866486; doi:10.1038/s41398-022-01844-x)
Supplement: Supplementary file 3 — Supplementary Fig. 3. [file 41398_2022_1844_MOESM3_ESM.pdf]

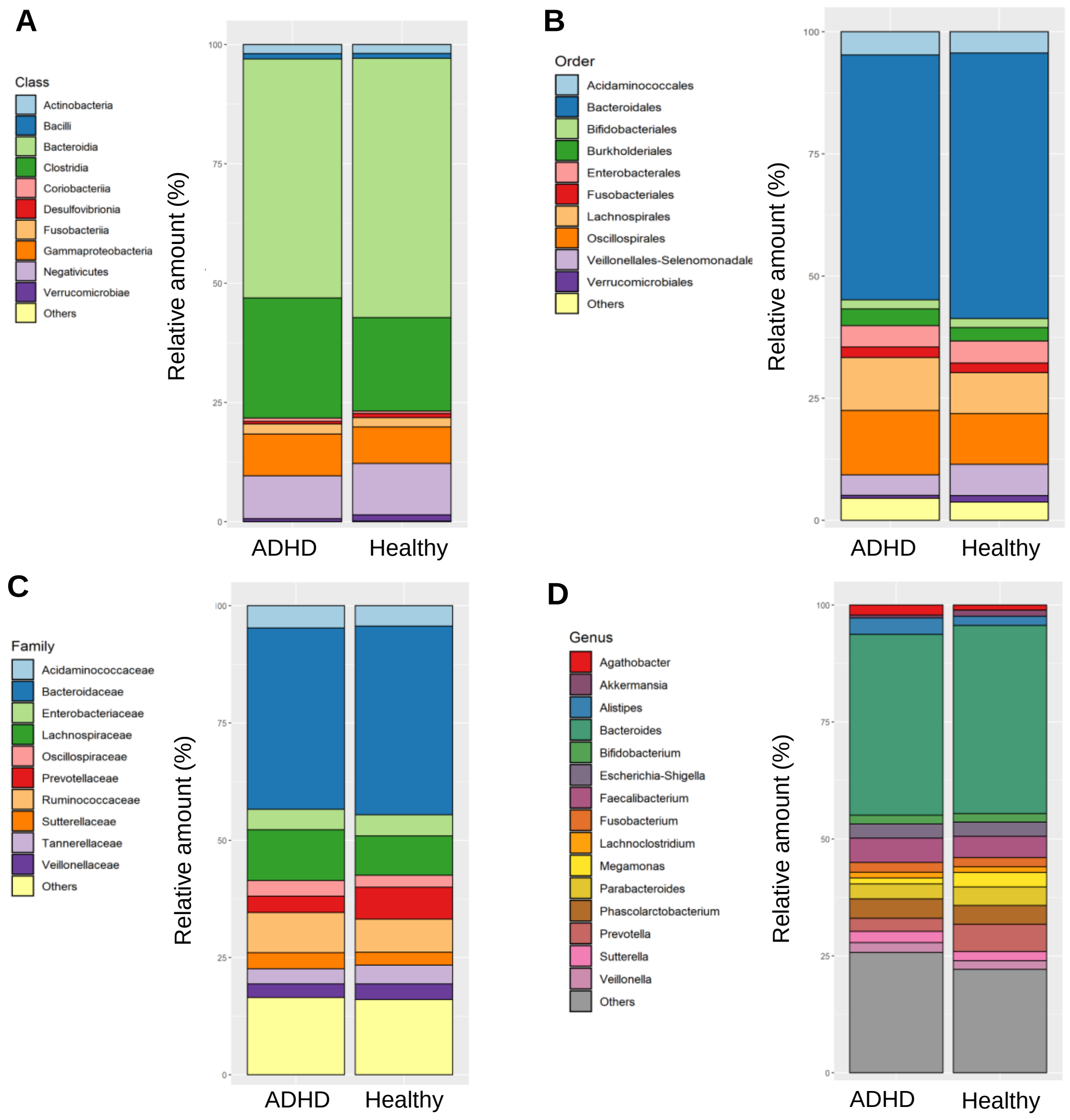

Supplementary Fig. 3. The bacterial taxonomic distributions from class to genus levels in ADHD and healthy controls. The most abundance class, order, family and genus were *Bacteroidia, Bacteroidales, Bacteroidaceae* and *Bacteroides*, respectively.
