# Supplementary material for: Gut microbiota and plasma cytokine levels in patients with attention-deficit/hyperactivity disorder
Source: Transl Psychiatry. 2022 Feb 23;12:76. doi: 10.1038/s41398-022-01844-x (PMC8866486; doi:10.1038/s41398-022-01844-x)
Supplement: Supplementary file 4 — Supplementary Fig. 4. [file 41398_2022_1844_MOESM4_ESM.pdf]

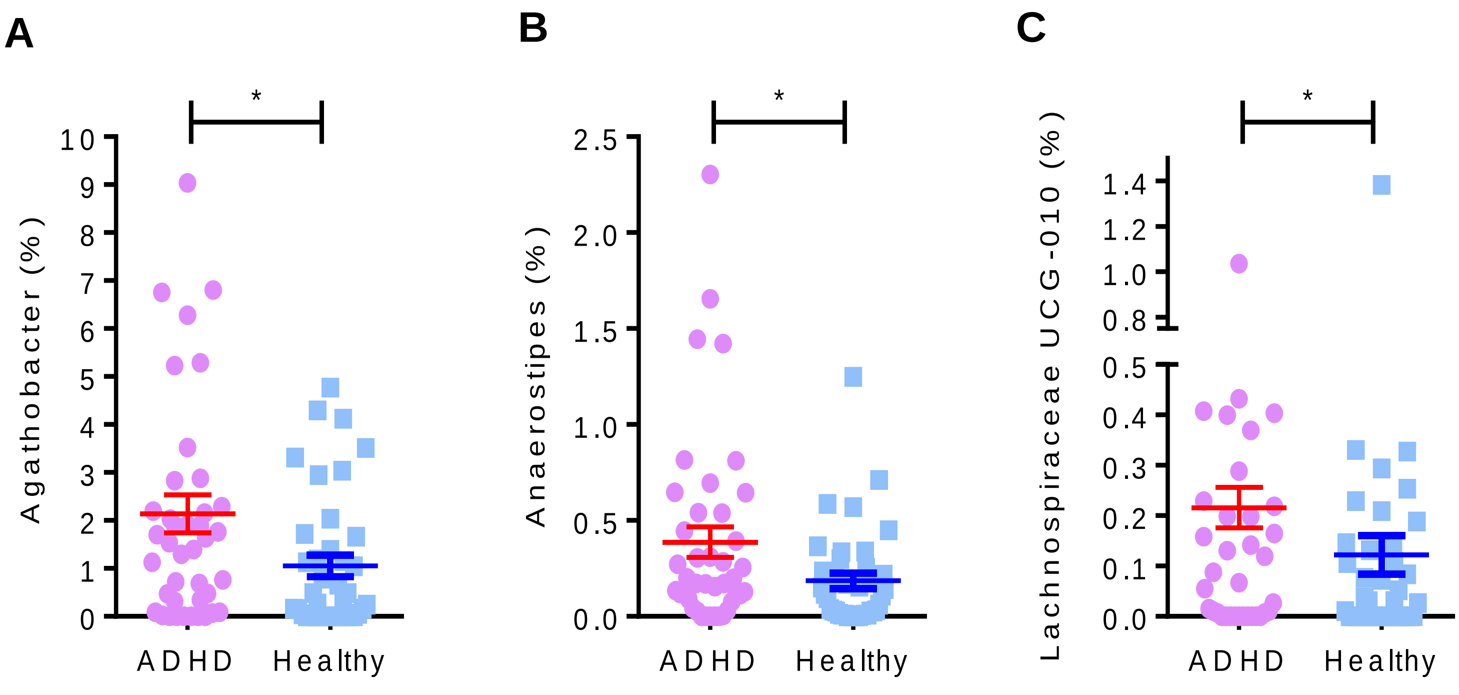

Supplementary Fig. 4. The bacterial taxonomy from family to genus levels in ADHD and healthy controls. The relative abundance of Agathobacter **(A)**, Anaerostipes **(B)**, and Lachnospiraceae UCG-010 **(C)** was shown in ADHD and healthy control group. The p-value was determined using analysis of covariance (ANCOVA) following adjustment with age and sex.
